# Assessing the relative importance of vitamin D deficiency in cardiovascular health

**DOI:** 10.3389/fcvm.2024.1435738

**Published:** 2024-10-16

**Authors:** Maira Rubab, John D. Kelleher

**Affiliations:** ^1^ Hamilton Institute, Maynooth University, Maynooth, Co. Kildare, Ireland; ^2^ ADAPT Research Centre, School of Computer Science and Statistics, Trinity College Dublin, College Green, Dublin, Ireland

**Keywords:** cardiovascular disease (CVD), CVD risk, vitamin D deficiency, machine learning (ML), random forest (RF), shapley additive explanations (SHAP), prediction, risk factors

## Abstract

Previous research has suggested a potential link between vitamin D (VD) deficiency and adverse cardiovascular health outcomes, although the findings have been inconsistent. This study investigates the association between VD deficiency and cardiovascular disease (CVD) within the context of established CVD risk factors. We utilized a Random Forest model to predict both CVD and VD deficiency risks, using a dataset of 1,078 observations from a rural Chinese population. Feature importance was evaluated using SHapley Additive exPlanations (SHAP) to discern the impact of various risk factors on the model’s output. The results showed that the model for CVD prediction achieved a high accuracy of 87%, demonstrating robust performance across precision, recall, and F1 score metrics. Conversely, the VD deficiency prediction model exhibited suboptimal performance, with an accuracy of 52% and lower precision, recall, and F1 scores. Feature importance analysis indicated that traditional risk factors such as systolic blood pressure, diastolic blood pressure, age, body mass index, and waist-to-hip ratio significantly influenced CVD risk, collectively contributing to 70% of the model’s predictive power. Although VD deficiency was associated with an increased risk of CVD, its importance in predicting CVD risk was notably low. Similarly, for VD deficiency prediction, CVD risk factors such as systolic blood pressure, glucose levels, diastolic blood pressure, and body mass index emerged as influential features. However, the overall predictive performance of the VD deficiency prediction model was weak (52%), indicating the absence of VD deficiency-related risk factors. Ablation experiments confirmed the relatively lower importance of VD deficiency in predicting CVD risk. Furthermore, the SHAP partial dependence plot revealed a nonlinear relationship between VD levels and CVD risk. In conclusion, while VD deficiency appears directly or indirectly associated with increased CVD risk, its relative importance within predictive models is considerably lower when compared to other risk factors. These findings suggest that VD deficiency may not warrant primary focus in CVD risk assessment and prevention strategies, however, further research is needed to explore the causal relationship between VD deficiency and CVD risk.

## 1 Introduction

Cardiovascular disease (CVD) remains a leading cause of morbidity and mortality worldwide, representing a significant public health challenge in the 21st century (1). In 2019, approximately 17.9 million individuals lost their lives to CVDs, accounting for about 32% of the total global mortality according to the World Health Organization (WHO). Among these fatalities, the majority, specifically 85%, resulted from heart attacks and strokes. Globally, the countries with the highest rates of CVD are India and China (2). It is becoming increasingly prevalent in China, where it is the leading cause of death (3). With the relentless rise in the global prevalence of CVD-related conditions such as hypertension, atherosclerosis, heart failure, stroke, myocardial infarction, coronary artery disease (CAD), and peripheral artery disease (PAD), there is an urgent need to identify modifiable risk factors. These factors can be harnessed to reduce the burden of this devastating disease. Although the etiology of CVD is complex, certain risk factors contribute to these conditions, such as elevated blood pressure, physical inactivity, smoking, age, cholesterol levels, family history, and body mass index (BMI) (4). The primary behavioral risk factors for heart disease and stroke include tobacco use, excessive alcohol consumption, unhealthy diet, and lack of physical activity. These factors can lead to elevated levels of blood pressure, blood glucose, and blood lipids, as well as obesity, which can be used to identify individuals at higher risk of experiencing heart attack, stroke, heart failure, and related conditions.

In recent years, emerging research has highlighted the role of vitamin D, a fat-soluble hormone primarily known for its crucial role in calcium homeostasis and bone health, as a potential contributor to the complex network of factors involved in CVD pathogenesis (5). Based on the existing evidence, vitamin D deficiency is emerging as a significant novel risk factor for CVD, potentially contributing causally to its development (6). Globally, around 1 billion people suffer from severe vitamin D deficiency (7). The most reliable indicator of vitamin D levels in the body is the circulation of 25-hydroxyvitamin D [25(OH)D] (8). Vitamin D has been extensively studied in cardiovascular clinical settings, focusing on prevalent conditions such as coronary artery disease, heart failure, and atrial fibrillation, which are among the most common CVDs globally (9). Vitamin D deficiency is frequently observed as a comorbidity in these conditions and has been linked to unfavorable short-term and long-term outcomes. This has led to the consideration of vitamin D supplementation for the prevention and treatment of several CVDs, although further research is needed due to inconsistent findings.

Numerous studies have explored the influence of vitamin D on CVD (10–12). Several have found that low levels of vitamin D are associated with an increased risk of CVD and that vitamin D is involved in various physiological processes related to cardiovascular health (13, 14). While some studies report a significant association between vitamin D deficiency and CVD, others show no clear link and have shown limitations for vitamin D supplementation (15, 16). The overall evidence has not been entirely consistent, and there is a lot of debate still happening within the scientific community. The inconsistent results often arise from variations in study designs, populations, methodologies, and potential confounding factors such as lifestyle, diet, sun exposure, physical activity, age, diabetes, and other health conditions. Most studies in the literature have used statistical methods to analyze the relationship between vitamin D and CVD, assess CVD prevalence, explore dose-response relationships, predict CVD risk, evaluate the severity of vitamin D deficiency, and determine the significance of their relationship (17–19). While these studies have explored the pairwise relationship between CVD and vitamin D and adjusted for confounding factors, there remains a need to assess the importance of vitamin D in conjunction with other CVD risk factors and understand the strength of its association with CVD, whether strong or weak. Focusing solely on pairwise relationships may overlook important features that independently contribute to the outcome or fail to capture the combined effects of multiple features, especially in cases where the relationship between features is non-linear. Feature importance, which refers to the contribution of each feature to a model’s predictions (20), offers a more comprehensive view. It indicates how informative a particular feature is to the model’s outcome. Instead of examining pairwise relationships between features using statistical methods, which may only capture specific relationships between two features at a time, assessing feature importance provides a broader perspective on the overall impact of each feature on the outcome.

Machine learning (ML) algorithms have the ability to capture complex non-linear interactions among features, unlike traditional statistical models, which often assume linear correlations and may struggle to handle high-dimensional data (21). This capability enables ML models to identify even subtle but significant associations (either strong or weak) between risk factors and CVD, providing a more comprehensive understanding of disease etiology by extracting feature importance alongside predictions. Because CVD is becoming one of the biggest threats to human health, it is increasingly important to develop an efficient CVD prediction strategy that analyzes the importance of various CVD risk factors. Most current medical approaches focus on disease detection rather than prediction. If CVD could be predicted in advance, early intervention might reduce the disease’s impact. However, even though the medical field collects vast amounts of data on a daily basis, analyzing these massive datasets using traditional methods can be challenging. Recent research has shown that ML methods can produce better outcomes (22). To identify individuals who are at high CVD risk, ML has the potential to outperform clinical prediction models, which are essentially based on statistics (23). Numerous studies have indicated that ML models exhibit better performance in calibration & discrimination compared to statistical models (24–26). ML models have the potential to entirely upgrade our approach to risk prediction and may even take the place of traditional statistical regression models in different fields (27, 28). In order to properly implement preventive public health interventions, ML models can help identify new underlying patterns (29). Weng et al. (30) and Monteiro et al. (31) concluded that the prediction of CVD risk can be greatly enhanced using ML. These pieces of evidence show that ML is experiencing a significant surge in utilization, particularly within the medical domain. Learning from input data, often known as training data, and subsequently employing this acquired knowledge to predict forthcoming events using new data is its primary goal.

To the best of our knowledge, no comprehensive study has focused on feature importance in predicting CVD risk using ML, specifically focusing on the importance of vitamin D in interaction with other risk factors. The noteworthy study conducted by Wang et al. (17) on a Chinese rural population utilized statistical methods and emphasized the association between vitamin D levels and CVD risk. By taking into account some confounding factors, their conclusion highlights that vitamin D deficiency is linked with an increased CVD risk. While this study provides valuable insights, its cross-sectional nature may not fully reflect the dynamic changes in vitamin D status that occur over longer periods. This is crucial because, in long-term prospective cohort studies, the observed association between vitamin D levels and CVD risk can be underestimated due to regression dilution bias (a phenomenon where fluctuations in vitamin D levels over time reduce the apparent strength of the association) (32).

Despite these findings, the specific role of vitamin D deficiency in the multifactorial interplay of CVD risk remains underexplored. An open question here is: does vitamin D deficiency significantly impact CVD risk when considered alongside other predictive factors? If so, this would suggest that more attention should be paid to vitamin D in CVD prevention. Accordingly, we aim to conduct an in-depth analysis of feature importance in predicting CVD risk, mainly focusing on vitamin D deficiency, to illuminate its actual significance and strength of association (whether weak or strong) with CVD risk within the context of other features. Additionally, we plan to explore this relationship bidirectionally by predicting vitamin D deficiency along with the feature importance analysis, while keeping CVD as one of the predictors. This approach will provide deeper insights into their association and serve two main purposes. Firstly, it helps to clarify whether vitamin D deficiency shares similar risk factors with CVD, thereby addressing whether the previous results indicating an association between them stem from common risk factors. Secondly, given the medical importance of vitamin D deficiency, predicting its risk on its own holds practical value. By analyzing both directions, we aim to enhance our understanding of their interplay, providing insights into their association. Furthermore, we incorporate ablation studies to refine our analysis.

Moreover, the reported study by Wang et al. has demonstrated a nonlinear relationship between CVD risk and vitamin D levels, so our goal is to employ a supervised ML algorithm that can cope with non-linearity for CVD risk prediction. To approach CVD risk prediction as a classification problem, we employed a Random Forest (RF) model (33), an ensemble learning technique that combines multiple decision trees to make predictions. Previous research has consistently demonstrated that RF outperforms numerous other ML models, particularly in risk prediction tasks related to CVD (34–38). The evidence from the literature strongly supports the use of RF as a preferred ML model for CVD risk prediction, due to its superior performance and robustness across diverse datasets and scenarios (39).

Additionally, RF provides a built-in method known as “Gini importance”, which relies on Gini impurity (40), for assessing feature importance. While this method provides a ranking of features based on their importance, it cannot indicate the direction (positive/negative) of a feature’s impact and does not consider the intricate interactions between the features, limiting its interpretability. To address these issues, we utilized a novel method called “SHapley Additive exPlanations (SHAP)” which is based on SHAP values (41). Unlike traditional metrics, SHAP offers a deeper understanding of feature contributions by quantifying the significance of each feature along with their direction and providing insights into individual predictions. By explicitly considering feature interactions and evaluating all possible subsets of features, SHAP provide a more nuanced interpretation of model behavior.

The paper is structured as follows: Section 2. covers related works, while Section 3. contains materials and methods including data description and preprocessing, explanation of RF and SHAP, experimental setup, model validation, and evaluation metrics. Section 4. presents results, comprising predictive performance assessment, various SHAP methods, and ablation results. Section 5. addresses paper discussion, limitations, and future work, while conclusions are drawn in Section 6.

## 2 Related works

Different reliable databases and scholarly websites were thoroughly examined in order to do an extensive analysis of the existing literature for this work. The 2 principal sources were PubMed, and Google Scholar.

Previous research has extensively analyzed the relationship between CVD and vitamin D, though most of this work has focused on statistical analysis (6, 10–12, 14–16, 18, 19, 42, 43). While some studies have applied ML methods, these typically focus either exclusively on CVD or vitamin D. For example, the datasets used in (34–37, 44–49) lack features related to vitamin D, and even if ML models were employed for CVD risk prediction, there was no further description regarding feature importance. Sambasivam et al. (50) utilized multiple ML models to analyze the predictive performance of vitamin D deficiency severity and conducted a comparative analysis of these models. Similarly, Guo et al. (51) used ML to predict vitamin D status and compared the results from the models they used. However, feature importance analysis was not taken into consideration in both studies, and CVD was not included at all in their analysis.

The studies (52–54) compared various ML models for predicting CVD. While they conducted a brief analysis to identify the most contributing features, they did not utilize SHAP for a comprehensive feature analysis, as their primary focus was on analyzing the ML models. SHAP, however, provides deeper insights into the direction of feature relationships, feature interactions, and the relative as well as individual importance of each feature. Moreover, vitamin D was not included among the predictors in their analysis. However, these studies concluded a range of major risk factors depending on the models and datasets used. Some commonly identified factors included age, systolic blood pressure, and cholesterol.

The study (39) by Kim et al. used ML to explore the influence of vitamin D levels on the prediction of acute ischemic stroke. The study concluded that individuals with vitamin D deficiency were more likely to experience worse outcomes than those with higher vitamin D levels which might have certain associations with other predictive variables. Although a feature importance graph was created, it was not the main focus of their study.

In previous research, no study has utilized the same dataset to conduct a comprehensive analysis employing ML to predict CVD risk and assess relative feature importance by considering vitamin D as part of the feature analysis. Likewise, no study has conducted such analysis to predict the risk of vitamin D deficiency and assess relative feature importance, integrating CVD as part of the feature analysis.

## 3 Materials and methods

### 3.1 Data description and preprocessing

This study utilized the dataset obtained from (17), which was collected through questionnaires, blood reports, and physical & laboratory examinations of participants from Henan province, China, conducted in July & August of each year (2013–2015). It has a total of 1,078 observations with 32 features. In any scientific or analytical work, data serves as the primary building block. Without sufficient and appropriate data, it becomes challenging to draw meaningful conclusions. Data preprocessing is a crucial step in data analysis pipelines. It involves cleaning and preparing raw data before it is used for analysis. The first step is to address missing values. The dataset used in this study contained only a few missing values which we handled using mean imputation in continuous features and mode imputation in categorical features. Mean imputation involves replacing missing values with the mean of the respective feature while imputing with the mode that fills the missing values with the most frequent value in the feature (55). These imputations could potentially skew the data towards the central tendency. However, given that the features in our dataset demonstrated a normal distribution and had very few missing values, these imputations were considered appropriate, as they preserved the original structure and sample size of the dataset.

To gain insights into our analysis of the association between vitamin D deficiency and CVD risk, we transformed the original continuous feature “25(OH)D” into a categorical feature named Vitamin D “VD” with three categories based on 25-hydroxyvitamin D concentration level: Deficiency (<20 ng/ml), Insufficiency (≥20 ng/ml & ≤30 ng/ml), and Sufficiency (>30 ng/ml). The thresholds for these categories were taken from the study (14), and we will discuss them later in the discussion section to explain why we chose these.

We addressed the multicollinearity by examining pairwise correlations between independent features. An absolute threshold of 0.7 was used to determine correlated features. We then removed one feature from each correlated pair to reduce redundancy and improve the interpretability of our model. Features with stronger correlations to “CVD” within each correlated pair were retained to ensure that the features selected for analysis are more closely aligned with our predictive objectives.

Three features (HTN(hypertension), CHO/HF(coronary heart disease or heart failure), & STROKE) were removed, as they represented subcategories directly associated with CVD, and including them could potentially bias the prediction of CVD risk. Finally, we excluded “nation” from the analysis due to our focus on the Chinese population, as only three samples were from non-Chinese categories, making its inclusion irrelevant. The dataset has a total of 21 features after performing all of the above steps and is described completely in Table 1.

**Table 1:** Features of the dataset.

S/N	Attribute	Description	Type	Distribution
1	age	Age (years)	Continuous	59.78±11.80
			Categorical	
2	gender	Gender	1: Male	1: 428
			2: Female	2: 650
			Categorical	
3	edu	Education	1: Junior high school & below	1: 953
			2: High school & above	2: 125
			Categorical	
4	marry	Martial status	1: Married/Cohabitation	1: 932
			2: Single/Divorcement	2: 146
5	BMI	Body mass index (kg/m^2^)	Continuous	25.50±3.65
6	GLU	Glucose (mmol/l)	Continuous	6.23±2.77
7	TC	Total cholestrol (mmol/l)	Continuous	4.68±1.01
8	TG	Triglycerides (mmol/l)	Continuous	1.83±1.38
9	HDL	High density lipoprotein (mmol/l)	Continuous	1.24±0.32
10	INS	Insulin (μU/mL)	Continuous	13.30±7.83
			Categorical	
11	VD	Vitamin D levels	1: Deficiency	1: 587
			2: Insufficiency	2: 311
			3: Sufficiency	3: 180
12	WHR	Waist-to-hip ratio	Continuous	0.91±0.07
			Binary	
13	Salt	Salt intake	0: No	0: 891
			1: Yes	1: 187
			Binary	
14	tea	Tea intake	0: No	0: 941
			1: Yes	1: 137
			Categorical	
15	Activity	Physical activity	1: Mild	1: 475
			2: Moderate	2: 182
			3: Severe	3: 421
16	SBP	Systolic blood pressure (mm Hg)	Continuous	131.35±18.92
17	DBP	Diastolic blood pressure (mm Hg)	Continuous	80.72±10.51
			Binary	
18	T2DM	Presence of type 2 diabetes mellitus	0: No	0: 728
			1: Yes	1: 350
			Categorical	
19	occupation	Occupation	1: Factory worker	1: 90
			2: Agriculture & related worker	2: 915
			3: Administrator/manager	3: 73
			Binary	
20	high-fat	High fat intake	0: No	0: 877
			1: Yes	1: 201
			Binary	
21	CVD	Cardiovascular disease presence	0: No	0: 484
			1: Yes	1: 594

In order to prepare the categorical features for analysis, we used one-hot encoding, the most widely used approach in ML to encode categorical data (56). It transforms the categorical features into a binary format where each unique category becomes a separate binary column, to maintain the distinctiveness of categories without imposing ordinal relationships.

### 3.2 Random forest model

RF starts by generating multiple bootstrap samples from the original dataset (33). This process involves resampling the data with replacement, thereby maintaining the sample size while creating diverse subsets for training each decision tree. During the construction of each decision tree, a unique subset of features is randomly selected from the full feature set and utilized at every split node within that tree. This random feature selection process occurs independently for each tree, ensuring that different decision trees within the ensemble utilize distinct subsets of features. Consequently, this enhances the diversity among individual trees and reduces their correlation. Each decision tree is trained on a bootstrap sample using its specific subset of features. This step involves recursively partitioning the feature space, starting from the root node and continuing to the leaf nodes where no further splits are made. The objective is to improve the homogeneity of the subsets created at each node by selecting the feature and threshold value that either minimizes impurity or maximizes information gain. Impurity measures the mixedness of classes within a subset, while information gain quantifies the reduction in uncertainty about the class labels achieved by splitting the data based on a particular feature. In classification tasks, the aim is to assign a class label to each data instance based on its features. Each decision tree makes its prediction, and the ensemble prediction is determined by a majority voting scheme (aggregation) after all decision trees have made their predictions. Each decision tree votes for its predicted class label, and the final prediction is the class label that receives the most votes among all decision trees. This approach reduces the risk of overfitting and biases by considering the opinions of multiple models trained on different subsets of data.

### 3.3 Shapley Additive Explanations (SHAP)

SHAP is a powerful method for interpreting the outputs of any ML model (RF in our case), offering both local and global explanations (41, 57). It uses cooperative game theory to compute SHAP values that provide insights into an instance’s output by evaluating the contribution of each input feature. These values identify which features matter most to the model and how they influence the output. In ML, every feature gets a SHAP value based on its contribution to the prediction and these values are calculated by considering every possible combination of features and assessing their marginal contributions (41). The impact of each feature on each final prediction, the relative importance of each feature, and the model’s dependence on feature interaction are all indicated by SHAP values. Positive SHAP values indicate that the presence of a feature increases the prediction, while negative values indicate the opposite. The magnitude of the SHAP value represents the impact of the feature on the prediction. Larger values imply stronger influence. Features with higher absolute SHAP values are more influential in determining the prediction outcome.

### 3.4 Experimental setup

We aimed to develop 2 binary classification RF models: one to predict “CVD” and the other to predict “VD deficiency”. While “VD” is basically a three-class categorical feature (see Table 1), we simplified the classification task for the VD Deficiency prediction by merging the “Insufficiency” and “Sufficiency” classes into a single category labeled as “0”, which contained 491 instances indicating the absence of VD deficiency. We retained the “Deficiency” class as “1”, comprising 587 instances for VD deficiency prediction. This conversion allowed us to treat the problem as a binary classification task, aligning with our primary objective of investigating the relationship between VD deficiency and CVD. However, we retained “VD” with its 3 categories for CVD prediction.

We utilized the same validation techniques and evaluation metrics to assess the predictive performance of both models, while also employing the same SHAP methods for feature importance analysis. The SHAP methods used in our study involved beeswarm, dependence, and global bar plots. Additionally, ablation studies were conducted for each prediction experiment with a consistent setup: we systematically removed the “VD_Deficiency” feature for the CVD prediction experiment and the “CVD” feature for the VD deficiency prediction experiment, aiming to reveal their individual impact to the predictive performance of the respective models. The proposed workflow for our study is shown in Supplementary Figure S1.

### 3.5 Model validation

We initially split the dataset randomly into two parts: 80% for training and 20% for testing. Using the training set, we trained our RF models, fine-tuned their hyperparameters, and evaluated their predictive performance. For hyperparameter tuning, we employed Grid Search with Cross-Validation (GSCV) to ensure robustness and prevent overfitting. Although the dataset showed only a slight imbalance, we used stratified cross-validation as a precautionary measure.

Grid search iterates through all possible combinations of hyperparameter values covered by the specified grid, evaluating each combination to determine the optimal hyperparameter set based on a specified evaluation metric. Specifically, it involves defining a grid of hyperparameter values to search over (same for both models), and training and evaluating the models using cross-validation for each combination within this grid. In our case, we employed a 5-fold stratified CV, partitioning the training dataset into five equally sized subsets while preserving the distributions of target classes. We then iterated over five distinct combinations of training and validation subsets, training the models on four-fifths of the data and evaluating their performance on the remaining one-fifth. Through this iterative process, we obtained robust estimates of the performance of our models while minimizing bias and variance. Finally, we assessed the performance of our models on the test set to validate their ability to generalize to new, unseen data. Table 2 displays the hyperparameter values utilized for GSCV, including the specifics of the optimal hyperparameter set for both models.

**Table 2:** Tested and optimal hyperparameters for both models.

Hyperparameters	For CVD Prediction	For VD Deficiency Prediction
Tested	n_estimators = [20,60,100,120], max_features = [0.2,0.6,1.0], max_depth = [2,4,8], min_samples_split = [10,30,60], min_samples_leaf = [5,10,20]	
Optimal	n_estimators : 100, max_features : 0.2, max_depth : 8, min_samples_split : 30, min_samples_leaf : 10	n_estimators : 20, max_features : 0.2, max_depth : 8, min_samples_split : 10, min_samples_leaf : 20

### 3.6 Evaluation metrics

In this study, we performed a comprehensive evaluation of our predictive RF models employing established metrics such as accuracy, precision, recall, and F1 score. Accuracy measures the overall correctness of model predictions, while precision indicates the model’s ability to avoid false positives by calculating the proportion of true positives among all positive predictions. We also assessed recall, also known as sensitivity, which measures the model’s capability to capture relevant instances by correctly identifying the proportion of true positives among all actual positives. Additionally, we utilized the F1 score, a harmonic mean of precision and recall, to provide a balanced assessment of model performance, considering both false positives and false negatives.

We performed GSCV for all four evaluation metrics. Precision, recall, and F1 score metrics were assessed with the minority class designated as the positive class due to the slight data imbalance. The best hyperparameter set was selected based on the accuracy metric, prioritizing the set with the highest mean accuracy across the 5 CV folds. Subsequently, we utilized this optimal hyperparameter set to retrain the models on the full training split of the data and then ran the retrained models on the test set to obtain the test scores for each metric.

## 4 Results

The data preprocessing, implementation of RF models, model validation, model evaluation, and SHAP analysis were executed in Python 3.10.9, utilizing libraries such as pandas, NumPy, Matplotlib, Seaborn, Scikit-learn, and SHAP. Next, we present an overview of the prediction performance achieved by the models proposed in our study, and discuss the feature importance using SHAP in predicting CVD risk and VD deficiency, focusing on the relationship of VD deficiency with CVD and their interaction with other features.

### 4.1 Predictive performance assessment

Table 3 presents the mean validation scores for all metrics across the 5 folds for the optimal hyperparameter set along with the test metric scores for both RF models.

**Table 3:** Summary of mean validation and test metric scores for both RF models with optimal hyperparameters.

	Metric	For CVD Target	For VD Deficiency Target
Mean validation scores	Accuracy	0.84±0.02	0.58±0.02
	Precision	0.80±0.04	0.56±0.03
	Recall	0.85±0.03	0.37±0.05
	F1 Score	0.82±0.02	0.45±0.04
Test scores	Accuracy	0.87	0.52
	Precision	0.85	0.46
	Recall	0.87	0.24
	F1 Score	0.86	0.32

#### 4.1.1 CVD prediction

The model achieved a mean accuracy of 84% during hyperparameter tuning, indicating that it could correctly classify individuals into CVD risk categories with high accuracy. The mean validation scores for precision, recall, and F1 score were 80%, 85%, and 82% respectively. These scores suggest that the model consistently performed well across different evaluation metrics during validation. The test scores further validate the robustness of this model, demonstrating its ability to generalize effectively to unseen data with an improved accuracy of 87%. Additionally, the precision, recall, and F1 score on the test set were all above 85%, demonstrating the model’s ability to correctly identify individuals at risk of CVD while minimizing false positives and false negatives. Overall, these results suggest the strong performance of this model, providing reliable predictions of CVD risk based on the independent features provided in Table 1.

#### 4.1.2 VD deficiency prediction

The predictive performance of this model appears to be less optimal (see Table 3). The best mean accuracy achieved during hyperparameter tuning was 58%, indicating that the model struggled to accurately classify individuals into VD deficiency categories. The mean validation scores for precision, recall, and F1 score were notably low, with values of 56%, 37%, and 45%, respectively. These scores suggest that this model exhibited less stability and poor overall performance during validation. On the test set, this model performed even worse with an accuracy of 52%, and all other metrics also showed a decrease in their scores, potentially indicating overfitting. This suggests that the independent features are not significantly contributing to the model’s performance, and it is very hard to predict VD deficiency with these predictors as they seem to be less relevant to VD deficiency than CVD.

The difference in performance between these two models indicates that predicting CVD risk based on the given dataset is more straightforward than predicting VD deficiency, and the feature set provided is better suited for modeling CVD risk than VD deficiency.

### 4.2 Global feature interpretation

Utilizing the SHAP global importance plot, we assessed the mean absolute value of each feature’s SHAP score across all observations within the dataset. This technique allowed us to quantify the influence of each feature on the model’s predictions, providing insights into their relative importance. By constructing stacked bar plots for each model based on their mean absolute values, we gained clear visualizations of the cumulative impact of each feature on the predictive performance of our models (see Supplementary Figures S2 and S3). These visualizations helped identify the top-ranking features, showing the factors that contributed most significantly to the outcomes of our models.

In Supplementary Figures S2 and S3, the x-axis shows the average impact of each feature on the model’s output, and the y-axis shows the top 15 features in descending order based on their importance, with the most influential features appearing at the top of the plot. The length of each bar indicates the average magnitude of the SHAP values for that feature across all observations. Longer bars represent features with greater influence on the model’s predictions, while shorter bars indicate lesser importance. The colors in the graph distinguish the categories of binary target features, with “blue” denoting “CVD” and “Deficient” class, and “red” indicating “non-CVD” and “non-Deficient” class for Supplementary Figures S2 and S3, respectively. Every bar is divided in half due to the binary nature of the targets, where the prediction of one category inherently explains the other. Supplementary Figure S2 illustrates a skewed distribution across the features which suggests variations in importance, with certain features exerting a more significant influence on the prediction outcome than others. In contrast, in Supplementary Figure S3, although there is still some skewness, the distribution appears slightly less pronounced. This implies that the importance of features is distributed somewhat more evenly, with fewer factors exerting an overly significant impact on the prediction outcome.

We present the top 15 features, each with their corresponding mean absolute SHAP values (summing from both classes), alongside their respective contributions as percentages to the overall feature score, for both models, in Table 4. The feature score is the sum of mean absolute SHAP values for each feature across all classes. The feature score for the CVD prediction model is 0.848, while for the VD deficiency prediction model, it is 0.363. The difference in feature scores between these two models indicates that the CVD prediction model relies more heavily on its top 15 features for making predictions compared to the VD deficiency prediction model.

**Table 4:** Mean absolute SHAP values & overall % contributions.

For CVD Prediction			For VD Deficiency Prediction		
Features	SHAP Scores	% Scores	Features	SHAP Scores	% Scores
SBP	0.2974	35.1	SBP	0.0690	19.0
DBP	0.1630	19.2	GLU	0.0326	9.0
age	0.0786	9.3	DBP	0.0322	8.9
WHR	0.0446	5.3	BMI	0.0306	8.4
BMI	0.0376	4.4	CVD	0.0292	8.0
INS	0.0330	3.9	TG	0.0192	5.3
GLU	0.0297	3.5	TC	0.0187	5.2
Acitivity_3	0.0250	3.0	HDL	0.0182	5.0
VD_Deficiency	0.0238	2.8	age	0.0178	4.9
Acitivity_1	0.0228	2.7	INS	0.0161	4.4
TG	0.0179	2.1	gender_1	0.0122	3.4
T2DM	0.0159	1.9	Activity_1	0.0121	3.3
VD_Insufficiency	0.0117	1.4	WHR	0.0112	3.1
HDL	0.0086	1.0	Activity_3	0.0088	2.4
gender_1	0.0074	0.9	high-fat	0.0082	2.3

For CVD prediction (see Supplementary Figure S2), the feature “SBP” emerges as the most influential predictor, contributing 35.1% to the overall prediction of CVD risk, which means that “SBP” plays a crucial role in determining an individual’s susceptibility to CVD. Focusing on the top 5 contributing features, namely “SBP”, “DBP”, “age”, “WHR”, and “BMI”, these features collectively account for approximately 70% of the prediction. This highlights the significance of traditional risk factors such as blood pressure, age, and obesity-related measures in predicting CVD risk. “VD_Deficiency” ranks among the top 10 contributors in CVD risk prediction. While its rank apparently suggests significance, its overall contribution of just 2.8% with SHAP score of 0.0238 is relatively low, indicating a weak relationship with CVD. Although “VD_Deficiency” shows an association with CVD, it may not be as important as other highly related features when considered alongside them.

For VD deficiency prediction (see Supplementary Figure S3), similar to CVD prediction, “SBP” emerges as the most influential predictor for VD deficiency, contributing 19.0% to the overall prediction. Analyzing the top 5 contributors, “SBP”, “GLU”, “DBP”, “BMI”, and “CVD” collectively contribute to approximately 50% of the prediction. Interestingly, while these factors are primarily CVD risk factors, they also show relevance to VD deficiency. Notably, “CVD” is among the top contributors, suggesting a potential bidirectional relationship between CVD and VD deficiency. However, despite “CVD” ranking fifth in this model, its overall contribution is relatively low compared to the topmost feature “SBP”. The low overall feature score of this model indicates that the model itself is very weak, suggesting that the features are not providing substantial assistance in predicting VD deficiency. Therefore, “CVD” may not be an important feature for predicting VD deficiency, even though it is associated with it.

Overall, the observed weak relationship between CVD and VD deficiency may arise from a multifactorial interplay among various predictive factors. It is plausible that the association between CVD and VD deficiency is not purely causal but rather influenced by complex interactions involving multiple physiological and environmental variables. Interestingly, the top five predictive features identified in both the CVD and VD deficiency prediction models overlap significantly. This overlap raises the possibility that these factors, such as blood pressure, glucose, age, and obesity-related measures, may have direct or indirect relationships with both VD deficiency and CVD risk. Therefore, while VD deficiency is associated with CVD, its significance in predicting CVD risk may be suppressed by its interactions with the shared major predictive factors.

### 4.3 SHAP beeswarm plot

The SHAP beeswarm plot is a scatter plot used to visualize the distribution of SHAP values for each feature across all observations in a dataset. In Supplementary Figures S4 and S5, each data point within the plot represents an individual instance from the test set. Its position along the x-axis indicates both the magnitude and direction of the corresponding feature’s SHAP value and the y-axis displays the top 15 features arranged in descending order of importance. A SHAP value of “0” signifies no contribution to the model’s prediction. Values to the right (positive) suggest the feature increases the likelihood of the model predicting the presence of CVD or VD deficiency. Conversely, values to the left (negative) indicate a decrease in the likelihood of predicting CVD or VD deficiency. The magnitude of the SHAP values tells the degree of influence each feature observation has on the model’s prediction outcome. The color scale on the right indicates the actual value of the feature for each observation, with “High” representing high values (which can mean high numerical values, presence/absence for binary features, or one-hot encoded categories) and “Low” representing low values.

Horizontal dispersion of points reveals the variability of SHAP values across observations, indicating patterns of feature importance and their interactions. Features with wider distributions suggest greater variability in their impact on model predictions, while those with narrower distributions exhibit more consistent effects. Influential features significantly affecting model predictions can be identified by observing the spread and concentration of points. A widespread or clustering of points at extreme ends of the plot typically indicates high importance. Additionally, features associated with consistent patterns of large positive or negative SHAP values across observations are considered influential.

Supplementary Figure S4 illustrates the distribution of SHAP values for predicting the CVD class. Conversely, the SHAP Beeswarm plot for the non-CVD class reveals an opposite pattern. “SBP” has a concentration of red dots to the extreme right, suggesting that higher values of “SBP” have a strong positive impact on predicting CVD. “DBP” has a similar distribution to “SBP” showing as the value of “DBP” increases (moving from blue to red), the impact on the model’s prediction shifts from lowering to increasing the risk of CVD. “age” has red dots mostly on the positive side, indicating that older individuals are at higher risk of CVD. Elevated values of “BMI” and “WHR” are similarly associated with a higher risk of CVD. The “VD_Deficiency” has SHAP values scattered across both sides, but with the cluster of red dots on the positive side, suggesting that when VD deficiency is true, it contributes to an increased risk of CVD prediction. However, the SHAP values for this feature are quite small (near 0), showing its minimal importance compared to the top-ranking features. Likewise, individuals who engage in mild physical activity (“Activity_1”) instead of moderate (“Activity_2”) and severe (“Activity_3”) are depicted as being at a higher risk of CVD.

Supplementary Figure S5 illustrates the distribution of SHAP values for predicting the VD deficient class. This graph also shows that higher SBP values contribute to a higher risk of vitamin D deficiency with a more consistent effect. “GLU” has a concentration of blue dots on the left with a slight progression towards the right side, implying an increased likelihood of having VD deficiency as glucose levels rise. Similarly, “DBP” appears to correlate higher DBP levels with an increased risk of VD deficiency. “BMI” and “age” display a blend of positive and negative SHAP values, indicating a more complex relationship with VD deficiency. Moreover, distinct clusters in CVD show that individuals with CVD are more likely to be at risk for VD deficiency.

### 4.4 Ablation results

In the previous section, we utilized SHAP to assess the relative importance of features. Another informative approach to understanding the importance of features involves conducting ablation studies. This technique helps evaluate the impact of individual features on the model’s performance by systematically removing specific features and observing changes in the model’s performance. By comparing the model’s performance with and without a particular feature, we can determine the feature’s influence on the model’s predictions. In our case, the feature of interest is “VD_Deficiency” in the CVD prediction model and “CVD” in the VD deficiency prediction model.

#### 4.4.1 CVD prediction

After excluding the “VD_Deficiency” feature from the analysis, the model achieved the following performance metrics on the test set: an accuracy of 0.86, precision of 0.83, recall of 0.86, and F1 score of 0.85. The test accuracy experienced a marginal decrease of 1%, in comparison to the test scores listed in Table 3, suggesting that the omission of the “VD_Deficiency” feature had a minor adverse effect on the model’s ability to accurately classify individuals into CVD risk categories. The test precision decreased by 2%, indicating a slight reduction in the model’s capability to avoid false positives. The test recall also remained almost the same, indicating that the model’s ability to capture relevant observations of CVD risk was largely unaffected by removing “VD_Deficiency” feature. Likewise, the F1 score decreased by 1%, indicating a slight decrease in the overall balance between precision and recall. This marginal difference in the model’s performance, ranging from just 1 to 2%, suggests that the inclusion or exclusion of the “VD_Deficiency” feature does not significantly impact the model’s effectiveness, implying a very weak association between VD deficiency and CVD.

#### 4.4.2 VD deficiency prediction

After excluding the “CVD” feature from the analysis, the model achieved the following performance metrics on the test set: an accuracy of 0.50, precision of 0.40, recall of 0.17, and F1 score of 0.24. Compared with the test scores listed in Table 3, there is a slight decrease of 2% in accuracy, while precision, recall, and F1 scores experienced larger decreases of 6%, 7%, and 8%, respectively. Although these differences suggest that the “CVD” feature may contribute to the model’s performance to some extent, the overall poor performance of the model indicates that the observed changes in metrics lack meaningful practical significance.

## 5 Discussion

In our analysis, we examined the importance of various features. Our findings highlight that both “SBP” and “DBP” play the most important role in predicting CVD, with “SBP” being the major risk factor for CVD followed by “DBP”. This result is in line with earlier findings reported in studies (58, 59). The high importance of “age” also aligns with the well-established understanding that CVD risk tends to rise with age, as noted in (30). Additionally, “WHR” and “BMI” are recognized markers of obesity, a known CVD risk factor (60). The top 10 features identified in our analysis largely coincide with recent research outlining 10 key CVD risk factors (61). Notably, “VD_Deficiency” appears to have low significance in predicting CVD risk and a weak relationship with an increased CVD risk. The lack of substantial evidence linking VD deficiency to CVD risk is notable, as other factors such as age, obesity-related measures, elevated blood pressure, physical inactivity, high cholesterol, etc. are consistently highlighted across studies. This observation is consistent with information from various studies and reputable sources like the World Health Organization (WHO), the Centers for Disease Control and Prevention (CDC), the National Health Service (NHS), and the American Heart Association (AHA) (61, 62). The strong predictive performance of our CVD risk prediction model, along with the evidence from past research, enhances the reliability of our results. Our ablation results further confirm the relatively lower importance of VD deficiency compared to other significant factors in assessing CVD risk.

One important factor to consider is that VD levels are primarily influenced by solar UVB exposure, which not only drives VD synthesis but also increases serum nitric oxide (NO) levels, a compound known to have cardiovascular benefits, such as lowering blood pressure (63, 64). This suggests that VD levels may serve as an index for both VD and NO. Consequently, the weak association observed between VD deficiency and CVD risk may be due to the broader cardio-protective effects of sunlight exposure through nitric oxide production. This highlights the complex interplay between sunlight, VD, and NO in maintaining cardiovascular health (65, 66).

The analysis of feature importance in predicting VD deficiency suggests the relevance of CVD risk factors namely, “SBP”, “GLU”, “DBP”, and “BMI”. Our results indicate that higher values of these key features are associated with an increased risk of VD deficiency. While existing literature suggests some associations between VD deficiency and these features (67–69), it typically discusses the association by considering the target and risk factors in reverse; that is, VD deficiency is associated with an increased risk of these features. However, the significance and direction of these relationships still require further investigation due to inconsistent findings, for instance, in the study (70). Given the potential interaction of VD deficiency with CVD risk factors, it is evident that an association between VD deficiency and CVD exists. Our results confirm the direction of these relationships. However, due to the poor performance of this model, the significance of the true relationship of these features (including CVD) with VD deficiency diminishes. VD deficiency can be influenced by a wide range of risk factors beyond those listed, including sunlight exposure, dietary intake of VD, seasonal variations, and more (69, 71). If these critical predictors are not included or accurately captured in the model, it may fail to fully comprehend the underlying mechanisms leading to VD deficiency, thereby impacting its performance. Even the predictors that we included and are known to be associated with VD deficiency in the literature, such as age and gender, are assigned very low ranks in our results. This may be due to the presence of top-ranked features that have some association but are not very important in VD deficiency prediction.

A potential confounding factor for our analysis is that we created the VD feature by categorizing the continuous feature ”25(OH)D”. This feature transformation may have affected the results we obtained. To check whether this occurred we conducted another experiment to predict CVD, maintaining the VD feature in its original continuous form, denoted as “25(OH)D”, while keeping the same settings as in our previous CVD prediction model. The objective was to examine the individual relationship between “25(OH)D” and CVD risk and to verify whether the chosen thresholds (same as given by the study (17)) to categorize this feature was appropriate. From this experiment, we generated a SHAP partial dependence plot. A SHAP partial dependence plot illustrates the marginal effect of an individual feature on the model’s prediction while accounting for the average effect of all other features. This isolates the influence of the analyzed feature on the model’s predictions, offering a clear visualization of its direct impact. Moreover, this plot reveals the nature of the relationship, whether it’s linear, monotonic, or exhibits more complex dynamics.

Supplementary Figure S6 illustrates the relationship between “25(OH)D”, shown on the x-axis, and its SHAP values, represented on the y-axis, with a color gradient indicating “SBP”. In this plot, we observe that lower values of “25(OH)D” (towards the left of the plot) are associated with more positive SHAP values, suggesting that lower VD levels increase the likelihood of the model predicting the positive class (CVD). Our threshold for defining VD deficiency is set at levels below 20. Notably, as VD levels approach 20, SHAP values begin to decrease. When they exceed 20, SHAP values increasingly become negative, indicating a decrease in the likelihood of the model predicting the positive class.

It is noted in (72) that the risk of CVD increases rapidly below 20. Therefore, using 20 as the threshold for VD deficiency may potentially underestimate the association between vitamin D deficiency and CVD risk. This observation aligns with the nonlinear relationship we observe in our data, particularly evident in Supplementary Figure S6, which suggests that exploring associations at lower thresholds may provide additional insights. Overall, the graph depicts a nonlinear relationship between “25(OH)D” and CVD risk, consistent with the findings of the study cited (17). Although the 20 threshold is commonly used and supported by the Endocrine Society (73), it may be useful to further investigate lower thresholds for enhanced understanding of the relationship between VD and CVD.

In summary, the association between VD deficiency and CVD exists, whether directly or indirectly, but the relative strength of this association is very weak. This implies that VD deficiency may not be a significant factor when interacts with other major CVD risk factors. Our findings indicate this weak association based on the relative importance of the features. Although there may be a causal association, the results are inconsistent in the literature. It remains unclear whether low VD levels directly cause CVD. The relationship between VD and the onset and progression of CVD is not well understood; it may simply reflect other health factors that are causally linked to the risk of CVD (74, 75). Additionally, VD’s causative role in CVD etiology has not been validated by Mendelian randomization studies (76, 77).

While this study offers insights into the relationship between CVD risk and VD deficiency within the Chinese population, it is important to acknowledge its limitations. Firstly, the cross-sectional nature of the study restricts the ability to explore the causal relationships. Additionally, the study’s focus on a specific geographic location and population may constrain the applicability of its findings to broader populations with diverse demographic profiles, varied environmental exposures, and different healthcare systems. Our future research will focus on acquiring longitudinal data to gain deeper insights into the causal dynamics between VD deficiency and various health outcomes, such as CVD. Furthermore, we aim to explore the potential risk factors associated with VD deficiency and enhance the predictive performance of our VD deficiency prediction model. Finally, we plan to utilize newly available datasets from diverse populations to expand upon and compare our current research findings.

## 6 Conclusions

Our research suggests that while there exists an association between VD deficiency and increased CVD risk in the Henan province of China, this relationship appears to be relatively weak in terms of its importance within predictive models. It is possible that this association between CVD and VD deficiency is not purely causal but is rather influenced by complex interactions of VD deficiency with the other CVD risk factors. Our findings suggest that VD deficiency does not exert a significant impact on CVD risk when compared to other well-established risk factors. Therefore, it may not warrant special attention as a primary focus in CVD risk assessment and prevention strategies. However, further investigation is important to validate these findings by exploring the causal relationship between VD deficiency and CVD risk, clarifying whether VD deficiency directly influences the development or progression of CVD.

## Data Availability

The original contributions presented in the study are included in the article/Supplementary Material, further inquiries can be directed to the corresponding author/s.

## References

[1] Case A Deaton A. Mortality and morbidity in the 21st century. Brook Pap Econ Act. (2017) 2017:397. 10.1353/eca.2017.0005 PMC5640267 29033460

[2] Zhao D Liu J Wang M Zhang X Zhou M. Epidemiology of cardiovascular disease in China: current features and implications. Nat Rev Cardiol. (2019) 16:203–12. 10.1038/s41569-018-0119-4 30467329

[3] Liu S Li Y Zeng X Wang H Yin P Wang L, et al. Burden of cardiovascular diseases in China, 1990–2016: findings from the 2016 global burden of disease study. JAMA Cardiol. (2019) 4:342–52. 10.1001/jamacardio.2019.0295 30865215 PMC6484795

[4] Bays HE Taub PR Epstein E Michos ED Ferraro RA Bailey AL, et al. Ten things to know about ten cardiovascular disease risk factors. Am J Prev Cardiol. (2021) 5:100149. 10.1016/j.ajpc.2021.100149 34327491 PMC8315386

[5] Murdaca G Gangemi S. Vitamin D in health and disease. Biomedicines. (2022) 11:10. 10.3390/biomedicines11010010 36672517 PMC9855922

[6] Anderson JL May HT Horne BD Bair TL Hall NL Carlquist JF, et al. Relation of vitamin D deficiency to cardiovascular risk factors, disease status, and incident events in a general healthcare population. Am J Cardiol. (2010) 106:963–8. 10.1016/j.amjcard.2010.05.027 20854958

[7] Holick MF. Vitamin D deficiency. N Engl J Med. (2007) 357:266–81. 10.1056/NEJMra070553 17634462

[8] Holick MF. Vitamin D status: measurement, interpretation, and clinical application. Ann Epidemiol. (2009) 19:73–8. 10.1016/j.annepidem.2007.12.001 18329892 PMC2665033

[9] Cosentino N Campodonico J Milazzo V De Metrio M Brambilla M Camera M, et al. Vitamin D and cardiovascular disease: current evidence and future perspectives. Nutrients. (2021) 13:3603. 10.3390/nu13103603 34684604 PMC8541123

[10] Wimalawansa SJ. Vitamin D and cardiovascular diseases: causality. J Steroid Biochem Mol Biol. (2018) 175:29–43. 10.1016/j.jsbmb.2016.12.016 28027913

[11] Thompson B Waterhouse M English DR McLeod DS Armstrong BK Baxter C, Vitamin D supplementation and major cardiovascular events: D-Health randomised controlled trial. BMJ. (2023) 381:e075230. 10.1136/bmj-2023-075230 37380191 PMC10302209

[12] Agarwal P Agarwal Y Hameed M. Recent advances in association between vitamin D levels and cardiovascular disorders. Curr Hypertens Rep. (2023) 25:185–209. 10.1007/s11906-023-01246-4 37256476

[13] Surdu AM Pînzariu O Ciobanu DM Negru AG Căinap SS Lazea C, et al. Vitamin D and its role in the lipid metabolism and the development of atherosclerosis. Biomedicines. (2021) 9:172. 10.3390/biomedicines9020172 33572397 PMC7916166

[14] Hung M Birmingham WC Ocampo M Mohajeri A. The role of vitamin D in cardiovascular diseases. Nutrients. (2023) 15:3547. 10.3390/nu15163547 37630735 PMC10459780

[15] Carvalho LSF Sposito AC. Vitamin D for the prevention of cardiovascular disease: are we ready for that? Atherosclerosis. (2015) 241:729–40. 10.1016/j.atherosclerosis.2015.06.034 26135478

[16] Manson JE Cook NR Lee IM Christen W Bassuk SS Mora S, et al. Vitamin D supplements and prevention of cancer and cardiovascular disease. N Engl J Med. (2019) 380:33–44. 10.1056/NEJMoa1809944 30415629 PMC6425757

[17] Wang T Sun H Ge H Liu X Yu F Han H, et al. Association between vitamin D and risk of cardiovascular disease in Chinese rural population. PLoS One. (2019) 14:e0217311. 10.1371/raj2Fjournal.pone.0217311 31120983 PMC6532968

[18] Sheehy S Palmer JR Cozier Y Bertrand KA Rosenberg L. Vitamin D and risk of hypertension among black women. J Clin Hypertens. (2023) 25:168–74. 10.1111/jch.14615 PMC9903189 36606491

[19] Mirhosseini N Rainsbury J Kimball SM. Vitamin D supplementation, serum 25 (OH) D concentrations and cardiovascular disease risk factors: a systematic review and meta-analysis. Front Cardiovasc Med. (2018) 5:87. 10.3389/fcvm.2018.00087 30050908 PMC6052909

[20] Fang F Ventre C Li L Kanthan L Wu F Basios M. Better model selection with a new definition of feature importance. *arXiv* [Preprint] *arXiv:2009.07708* (2020). Available online at: https://arxiv.org/abs/2009.07708 (accessed September 16, 2020).

[21] Kattan MW Hess KR Beck JR. Experiments to determine whether recursive partitioning (CART) or an artificial neural network overcomes theoretical limitations of Cox proportional hazards regression. Comput Biomed Res. (1998) 31:363–73. 10.1006/cbmr.1998.1488 9790741

[22] Lee Y Ragguett RM Mansur RB Boutilier JJ Rosenblat JD Trevizol A, et al. Applications of machine learning algorithms to predict therapeutic outcomes in depression: a meta-analysis and systematic review. J Affect Disord. (2018) 241:519–32. 10.1016/j.jad.2018.08.073 30153635

[23] Allan S Olaiya R Burhan R. Reviewing the use and quality of machine learning in developing clinical prediction models for cardiovascular disease. Postgrad Med J. (2022) 98:551–8. 10.1136/postgradmedj-2020-139352 33692158

[24] Steele AJ Denaxas SC Shah AD Hemingway H Luscombe NM. Machine learning models in electronic health records can outperform conventional survival models for predicting patient mortality in coronary artery disease. PLoS One. (2018) 13:e0202344. 10.1371/journal.pone.0202344 30169498 PMC6118376

[25] Alaa AM Bolton T Di Angelantonio E Rudd JH Van der Schaar M. Cardiovascular disease risk prediction using automated machine learning: a prospective study of 423,604 UK Biobank participants. PLoS One. (2019) 14:e0213653. 10.1371/journal.pone.0213653 31091238 PMC6519796

[26] Al’Aref SJ Anchouche K Singh G Slomka PJ Kolli KK Kumar A, et al. Clinical applications of machine learning in cardiovascular disease and its relevance to cardiac imaging. Eur Heart J. (2019) 40:1975–86. 10.1093/eurheartj/ehy404 30060039

[27] Hinton G. Deep learning—a technology with the potential to transform health care. JAMA. (2018) 320:1101–2. 10.1001/jama.2018.11100 30178065

[28] Makridakis S Spiliotis E Assimakopoulos V. Statistical and machine learning forecasting methods: concerns and ways forward. PLoS One. (2018) 13:e0194889. 10.1371/journal.pone.0194889 29584784 PMC5870978

[29] Alghunaim S Al-Baity HH. On the scalability of machine-learning algorithms for breast cancer prediction in big data context. IEEE Access. (2019) 7:91535–46. 10.1109/ACCESS.2019.2927080

[30] Tsao CW Aday AW Almarzooq ZI Alonso A Beaton AZ Bittencourt MS, et al. Heart disease and stroke statistics—2022 update: a report from the American Heart Association. Circulation. (2022) 145:e153–e639. 10.1161/CIR.0000000000001052 35078371

[31] Monteiro M Fonseca AC Freitas AT e Melo TP Francisco AP Ferro JM, et al. Using machine learning to improve the prediction of functional outcome in ischemic stroke patients. IEEE/ACM Trans Comput Biol Bioinform. (2018) 15:1953–9. 10.1109/TCBB.2018.2811471 29994736

[32] Clarke R Shipley M Lewington S Youngman L Collins R Marmot M, et al. Underestimation of risk associations due to regression dilution in long-term follow-up of prospective studies. Am J Epidemiol. (1999) 150:341–53. 10.1093/oxfordjournals.aje.a010013 10453810

[33] Biau G Scornet E. A random forest guided tour. Test. (2016) 25:197–227. 10.1007/s11749-016-0481-7

[34] Yang L Wu H Jin X Zheng P Hu S Xu X, et al. Study of cardiovascular disease prediction model based on random forest in eastern China. Sci Rep. (2020) 10:5245. 10.1038/s41598-020-62133-5 32251324 PMC7090086

[35] Kumar NK Sindhu GS Prashanthi DK Sulthana AS. Analysis and prediction of cardio vascular disease using machine learning classifiers. In: 2020 6th International Conference on Advanced Computing and Communication Systems (ICACCS). IEEE (2020). p. 15–21. 10.1109/ICACCS48705.2020.9074183

[36] Pal M Parija S. Prediction of heart diseases using random forest. In: Journal of Physics: Conference Series. Vol. 1817. IOP Publishing (2021). p. 012009. 10.1088/1742-6596/1817/1/012009

[37] Azmi J Arif M Nafis MT Alam MA Tanweer S Wang G. A systematic review on machine learning approaches for cardiovascular disease prediction using medical big data. Med Eng Phys. (2022) 105:103825. 10.1016/j.medengphy.2022.103825 35781385

[38] Rubini P Subasini C Katharine AV Kumaresan V Kumar SG Nithya T. A cardiovascular disease prediction using machine learning algorithms. Ann Roman Soc Cell Biol. (2021) 25:904–12.

[39] Kim C Lee SH Lim JS Kim Y Jang MU Oh MS, et al. Impact of 25-hydroxyvitamin D on the prognosis of acute ischemic stroke: machine learning approach. Front Neurol. (2020) 11:37. 10.3389/fneur.2020.00037 32082247 PMC7005206

[40] Louppe G Wehenkel L Sutera A Geurts P. Understanding variable importances in forests of randomized trees. Adv Neural Inf Process Syst. (2013) 26:431–9.

[41] Lundberg SM Lee SI. A unified approach to interpreting model predictions. Adv Neural Inf Process Syst. (2017) 30:4768–77. 10.48550/arXiv.1705.07874

[42] Cui A Xiao P Ma Y Fan Z Zhou F Zheng J, et al. Prevalence, trend, and predictor analyses of vitamin D deficiency in the US population, 2001–2018. Front Nutr. (2022) 9:965376. 10.3389/fnut.2022.965376 36263304 PMC9573946

[43] Bahrami LS Ranjbar G Norouzy A Arabi SM. Vitamin D supplementation effects on the clinical outcomes of patients with coronary artery disease: a systematic review and meta-analysis. Sci Rep. (2020) 10:12923. 10.1038/s41598-020-69762-w 32737345 PMC7395726

[44] Srinivasan S Gunasekaran S Mathivanan SK M B BAM Jayagopal P Dalu GT. An active learning machine technique based prediction of cardiovascular heart disease from UCI-repository database. Sci Rep. (2023) 13:13588. 10.1038/s41598-023-40717-1 37604952 PMC10442398

[45] Jadhav AD Chobe SV. Risk assessment of cardiovascular diseases using KNN and decision tree classifier. Rev d’Intell Artif. (2022) 36:155–61. 10.18280/ria.360118

[46] Princy RJP Parthasarathy S Jose PSH Lakshminarayanan AR Jeganathan S. Prediction of cardiac disease using supervised machine learning algorithms. In: 2020 4th International Conference on Intelligent Computing and Control Systems (ICICCS). IEEE (2020). p. 570–5. 10.1109/ICICCS48265.2020.9121169

[47] Balakrishnan M Christopher AA Ramprakash P Logeswari A. Prediction of cardiovascular disease using machine learning. In: Journal of Physics: Conference Series. Vol. 1767. IOP Publishing (2021). p. 012013. 10.1088/1742-6596/1767/1/012013

[48] Singh A Kumar R. Heart disease prediction using machine learning algorithms. In: 2020 International Conference on Electrical and Electronics Engineering (ICE3). IEEE (2020). p. 452–7. 10.1109/ICE348803.2020.9122958

[49] Pradhan M. Cardiovascular disease prediction using various machine learning algorithms. J Comput Sci. (2022) 18:993–1004. 10.3844/jcssp.2022.993.1004

[50] Sambasivam G Amudhavel J Sathya G. A predictive performance analysis of vitamin D deficiency severity using machine learning methods. IEEE Access. (2020) 8:109492–507. 10.1109/ACCESS.2020.3002191

[51] Guo S Lucas RM Ponsonby AL Group AI. A novel approach for prediction of vitamin D status using support vector regression. PLoS One. (2013) 8:e79970. 10.1371/journal.pone.0079970 24302994 PMC3841172

[52] Dinh A Miertschin S Young A Mohanty SD. A data-driven approach to predicting diabetes and cardiovascular disease with machine learning. BMC Med Inform Decis Mak. (2019) 19:1–15. 10.1186/s12911-019-0918-5 31694707 PMC6836338

[53] Peng M Hou F Cheng Z Shen T Liu K Zhao C, et al. Prediction of cardiovascular disease risk based on major contributing features. Sci Rep. (2023) 13:4778. 10.1038/s41598-023-31870-8 36959459 PMC10036320

[54] Weng SF Reps J Kai J Garibaldi JM Qureshi N. Can machine-learning improve cardiovascular risk prediction using routine clinical data? PLoS One. (2017) 12:e0174944. 10.1371/journal.pone.0174944 28376093 PMC5380334

[55] Aljuaid T Sasi S. Proper imputation techniques for missing values in data sets. In: 2016 International Conference on Data Science and Engineering (ICDSE). IEEE (2016). p. 1–5. 10.1109/ICDSE.2016.7823957

[56] Potdar K Pardawala TS Pai CD. A comparative study of categorical variable encoding techniques for neural network classifiers. Int J Comput Appl. (2017) 175:7–9. 10.5120/ijca2017915495

[57] Lundberg SM Erion G Chen H DeGrave A Prutkin JM Nair B, et al. From local explanations to global understanding with explainable AI for trees. Nat Mach Intell. (2020) 2:56–67. 10.1038/s42256-019-0138-9 32607472 PMC7326367

[58] Gu D Kelly TN Wu X Chen J Duan X Huang JF, et al. Blood pressure and risk of cardiovascular disease in Chinese men and women. Am J Hypertens. (2008) 21:265–72. 10.1038/ajh.2007.59 18188156

[59] Brunström M Carlberg B. Association of blood pressure lowering with mortality and cardiovascular disease across blood pressure levels: a systematic review and meta-analysis. JAMA Intern Med. (2018) 178:28–36. 10.1001/jamainternmed.2017.6015 29131895 PMC5833509

[60] Koliaki C Liatis S Kokkinos A. Obesity and cardiovascular disease: revisiting an old relationship. Metabolism. (2019) 92:98–107. 10.1016/j.metabol.2018.10.011 30399375

[61] Bays HE. Ten things to know about ten cardiovascular disease risk factors (“ASPC Top Ten–2020”). Am J Prev Cardiol. (2020) 1:100003. 10.1016/j.ajpc.2020.100003 34327447 PMC8315360

[62] Brown JC Gerhardt TE Kwon E. Risk Factors for Coronary Artery Disease. Treasure Island (FL): StatPearls Publishing (2020). http://europepmc.org/books/NBK554410 32119297

[63] Bryan NS. Nitric oxide deficiency is a primary driver of hypertension. Biochem Pharmacol. (2022) 206:115325. 10.1016/j.bcp.2022.115325 36349641

[64] Weller RB Wang Y He J Maddux FW Usvyat L Zhang H, et al. Does incident solar ultraviolet radiation lower blood pressure? J Am Heart Assoc. (2020) 9:e013837. 10.1161/JAHA.119.013837 32106744 PMC7335547

[65] Stevenson AC Clemens T Pairo-Castineira E Webb DJ Weller RB Dibben C. Higher ultraviolet light exposure is associated with lower mortality: an analysis of data from the UK Biobank cohort study. Health Place. (2024) 89:103328. 10.1016/j.healthplace.2024.103328 39094281

[66] Quan QL Yoon KN Lee JS Kim EJ Lee DH. Impact of ultraviolet radiation on cardiovascular and metabolic disorders: the role of nitric oxide and vitamin D. Photodermatol Photoimmunol Photomed. (2023) 39:573–81. 10.1111/phpp.12914 37731181

[67] Mozaffari-Khosravi H Loloei S Mirjalili MR Barzegar K. The effect of vitamin D supplementation on blood pressure in patients with elevated blood pressure and vitamin D deficiency: a randomized, double-blind, placebo-controlled trial. Blood Press Monit. (2015) 20:83–91. 10.1097/MBP.0000000000000091 25350782

[68] Valladares T Cardoso MR Aldrighi JM. Higher serum levels of vitamin D are associated with lower blood glucose levels. Menopause. (2019) 26:781–4. 10.1097/GME.0000000000001308 30694916

[69] Lagunova Z Porojnicu AC Lindberg F Hexeberg S Moan J. The dependency of vitamin D status on body mass index, gender, age and season. Anticancer Res. (2009) 29:3713–20. https://ar.iiarjournals.org/content/29/9/3713 19667169

[70] Tai K Need AG Horowitz M Chapman IM. Glucose tolerance and vitamin D: effects of treating vitamin D deficiency. Nutrition. (2008) 24:950–6. 10.1016/j.nut.2008.04.009 18653316

[71] Gonoodi K Tayefi M Saberi-Karimian M Darroudi S Farahmand SK Abasalti Z, et al. An assessment of the risk factors for vitamin D deficiency using a decision tree model. Diab Metab Syndr Clin Res Rev. (2019) 13:1773–7. 10.1016/j.dsx.2019.03.020 31235093

[72] Wang L Song Y Manson JE Pilz S März W Michaëlsson K, et al. Circulating 25-hydroxy-vitamin D and risk of cardiovascular disease: a meta-analysis of prospective studies. Circ Cardiovasc Qual Outcom. (2012) 5:819–29. 10.1161/CIRCOUTCOMES.112.967604 PMC3510675 23149428

[73] Holick MF Binkley NC Bischoff-Ferrari HA Gordon CM Hanley DA Heaney RP, et al. Evaluation, treatment, and prevention of vitamin D deficiency: an endocrine society clinical practice guideline. J Clin Endocrinol Metab. (2011) 96:1911–30. 10.1210/jc.2011-0385 21646368

[74] Pilz S Verheyen N Grübler MR Tomaschitz A März W. Vitamin D and cardiovascular disease prevention. Nat Rev Cardiol. (2016) 13:404–17. 10.1038/nrcardio.2016.73 27150190

[75] Lavie CJ Lee JH Milani RV. Vitamin D and cardiovascular disease: will it live up to its hype? J Am Coll Cardiol. (2011) 58:1547–56. 10.1016/j.jacc.2011.07.008 21958881

[76] Manousaki D Mokry LE Ross S Goltzman D Richards JB. Mendelian randomization studies do not support a role for vitamin D in coronary artery disease. Circ Cardiovasc Genet. (2016) 9:349–56. 10.1161/CIRCGENETICS.116.001396 27418593

[77] Afzal S Brøndum-Jacobsen P Bojesen SE Nordestgaard BG. Genetically low vitamin D concentrations and increased mortality: mendelian randomisation analysis in three large cohorts. BMJ. (2014) 349:g6330. 10.1136/bmj.g6330 25406188 PMC4238742

