# Supplementary figures and images for: Assessing the relative importance of vitamin D deficiency in cardiovascular health
Source: Front Cardiovasc Med. 2024 Oct 16;11:1435738. doi: 10.3389/fcvm.2024.1435738 (PMC11521893; doi:10.3389/fcvm.2024.1435738)

# Supplementary Material

## FIGURES

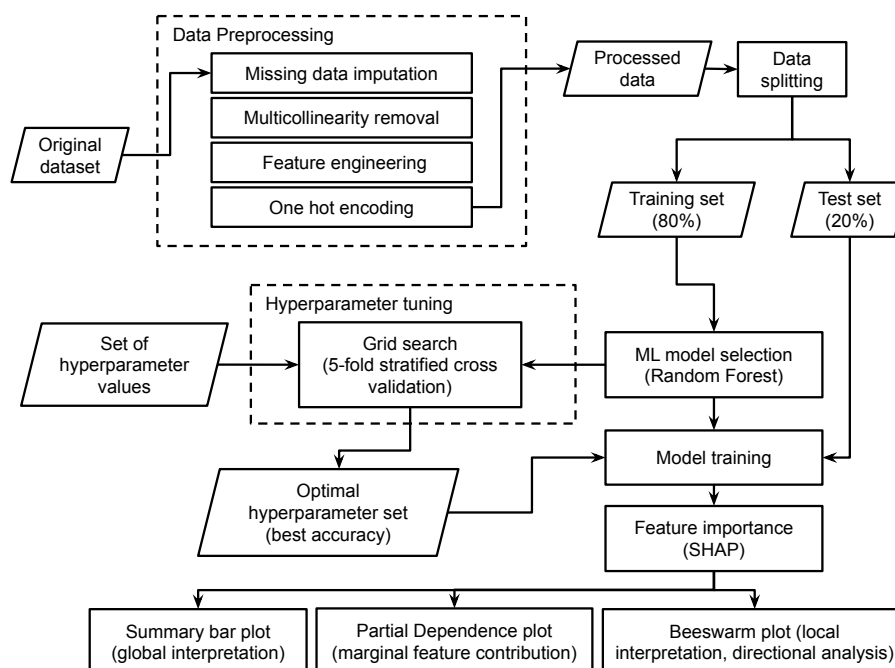

Figure S1.

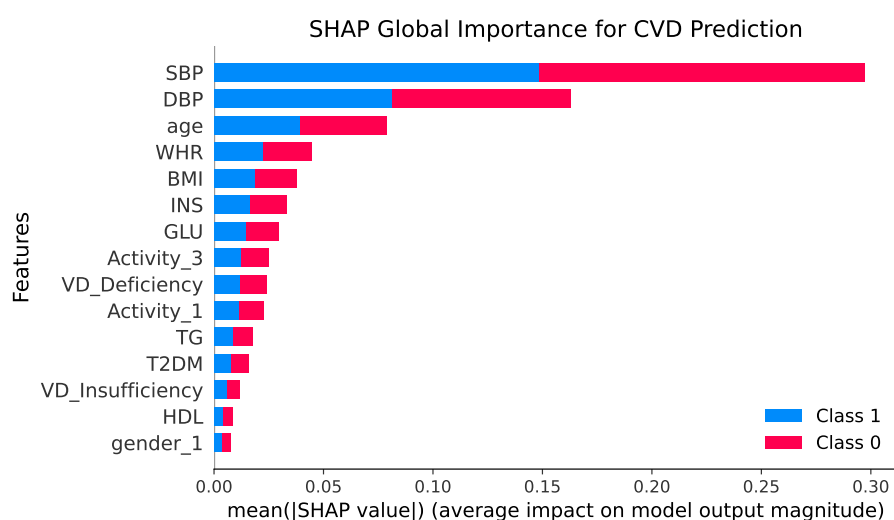

Figure S2.

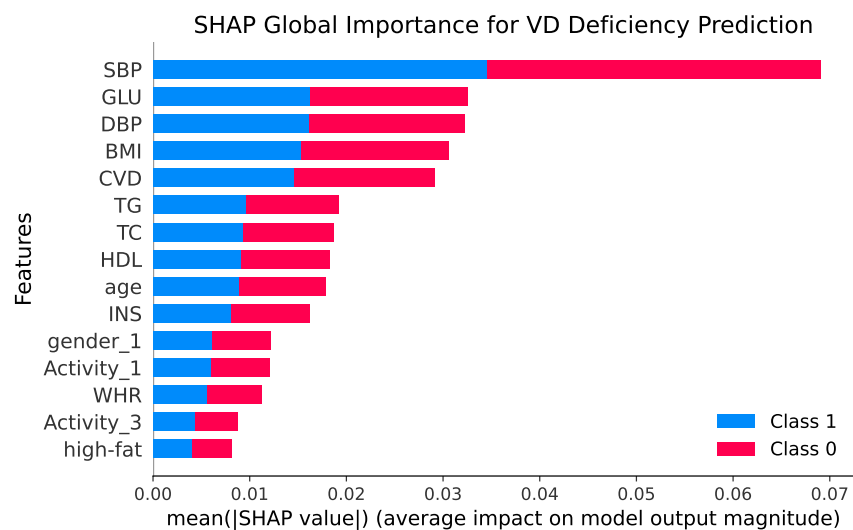

Figure S3.

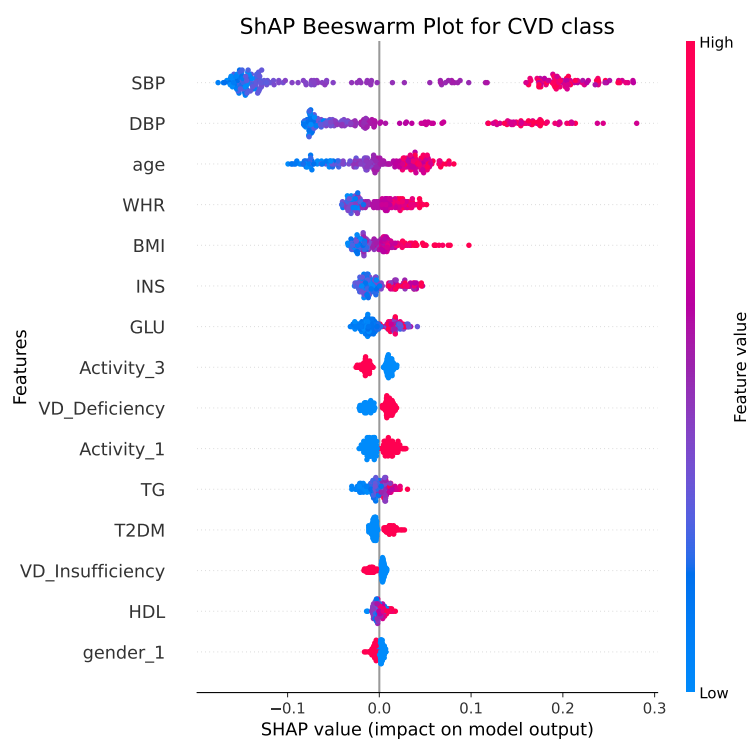

Figure S4.

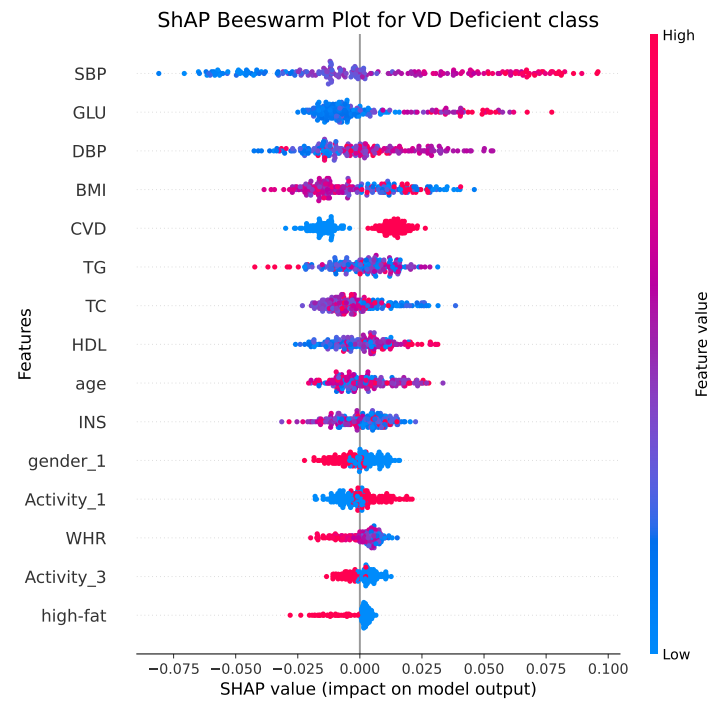**Figure S5.**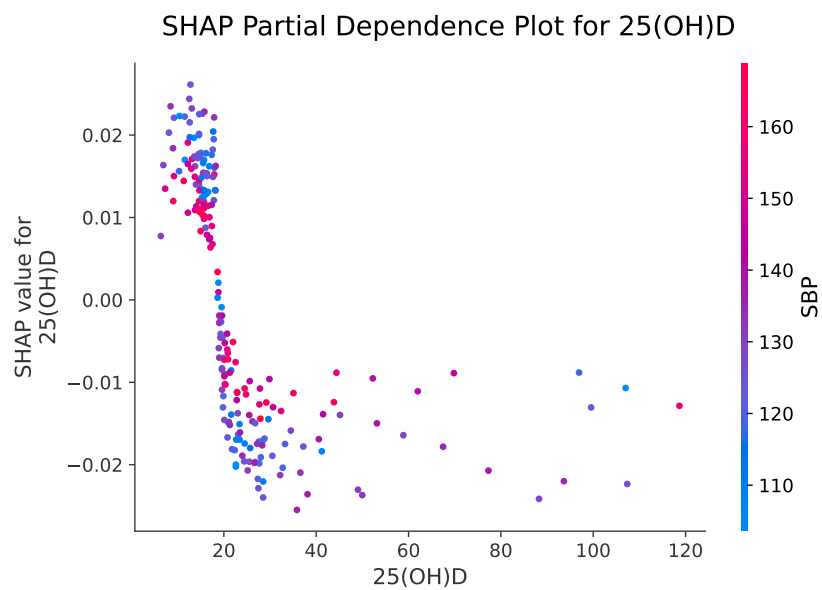**Figure S6.**

Supplement: Supplementary file 1 [file Datasheet1.pdf]
